# Metal health: PATELLIN2 reduces iron-induced toxicity in Arabidopsis

**DOI:** 10.1093/plphys/kiad090

**Published:** 2023-02-15

**Authors:** Stefanie Wege, José Manuel Ugalde

**Affiliations:** Plant Physiology, American Society of Plant Biologists, Rockville, MD, USA; Institute of Crop Science and Resource Conservation (INRES), University of Bonn, 53115 Bonn, Germany; Institute of Crop Science and Resource Conservation (INRES)—Chemical Signalling, University of Bonn, Friedrich-Ebert-Allee 144, 53113 Bonn, Germany

**Figure 1.**
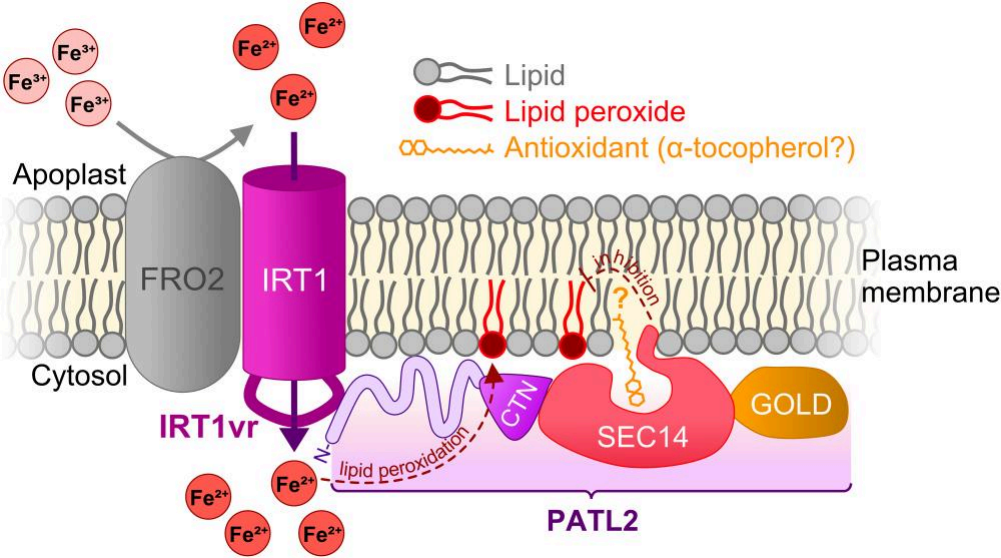
Model of PATL2-mediated reduction of Fe-induced oxidative damage. Apoplastic Ferric Fe (Fe^3+^) is reduced by membrane-bound FERRIC REDUCTION REDUCTASE 2 (FRO2) to ferrous Fe (Fe^2+^), which is transported to the cytosol via IRT1. Excess Fe^2+^ can interact with ROS leading to oxidative damage by promoting lipid peroxidation. The N-terminus of PATL2 (CTN-SEC14-GOLD) interacts with the variable region of IRT1 (IRT1vr), and its SEC14 domain binds α-tocopherol in vitro. The current model hypothesizes that PATL2 might make the antioxidant α-tocopherol available where lipid peroxidation is occurring, alleviating oxidative damage. Further work will be required to confirm if α-tocopherol or another component is binding to PATL2 in vivo (Modified figure from Hornbergs et al. (2023) made by J.M.U. using Affinity Designer 2 Version 2.0.3).

Iron (Fe) is an essential micronutrient for plants, which are in turn the main Fe source for most of the world's population (Brumbarova et al. 2015). Plants have evolved elaborate mechanisms for Fe uptake and re-distribution in which the plasma membrane (PM) transporter IRON REGULATED TRANSPORTER 1 (IRT1) plays a crucial role in Fe uptake from the soil (Vert et al. 2002) (Fig. 1). Yet, typical for divalent cation transporters, IRT1 is not Fe-specific and transports other divalent cations as well, such as cadmium. Additionally, Fe itself is dangerous to the cell due to its capacity to feed the Fenton reaction, which can have numerous negative consequences, including protein damage and lipid peroxidation (Gratz et al. 2021). This conundrum has led cells to establish a tight regulation of the transporter. For example, IRT1 is only recruited to the PM when Fe uptake takes place; otherwise, it resides in small compartments of the endomembrane system. The internalization, recycling, and degradation of IRT1 are controlled by ubiquitination at a large cytoplasmic loop known as the IRT1 variable region (IRT1vr) (Guerinot 2000). While the cell can tightly control IRT1 localization through this mechanism, once it is active on the PM, the cell has to deal with the uptake of dangerous Fe or even toxic cadmium. However, the IRT1-related regulatory mechanisms that prevent Fe-induced oxidative damage, and in particular lipid peroxidation, have remained unknown.

In this issue of *Plant Physiology*, Hornbergs et al. (2023) investigated PATELLIN2 (PATL2) as an IRT1 interaction partner. Using a combination of yeast two-hybrid assays, biomolecular fluorescence complementation, and co-immuno precipitation they showed that the N-terminal domain of PATL2 specifically interacts with IRT1vr.

PATL2 is a member of the PATL protein family characterized by three distinct subdomains. These domains, from PATL2 and other related proteins have been demonstrated to bind and deliver lipophilic molecules to membranes and enzymes and mediated protein–lipid interaction (de Campos and Schaaf 2017; Montag et al. 2020). Plants deficient in PATL2 showed higher ferric reductase activity and higher levels of peroxide lipids when compared to wild-type plants, indicating that PATL2 is needed to alleviate lipid peroxidation (Fig. 1). Through in vitro assays, Hornbergs et al. (2023) show that PATL2 can bind a vitamin E variant, α-tocopherol, and they used computational modeling to show that α-tocopherol could fit into the lipophilic pocket of PATL2 (Fig. 1). Vitamin E variants, such as α-tocopherol, are strong antioxidants particularly used in preventing lipid peroxidation. This might suggest that PATL2 delivers α-tocopherol directly to IRT1 to alleviate reactive oxygen species (ROS) damage directly during transport activity (Fig. 1). The role of PATL2 in ROS damage prevention is supported by the PATL2 interactome in which the authors identified many proteins known to contribute to antioxidant ROS-processing, such as glutathione peroxidases and glutathione S-transferases, the latter of which comprise a superfamily of enzymes with members already known to detoxify lipid peroxides (Ugalde et al. 2021).

The interaction of PATL2 with IRT1vr and the recruitment of antioxidants directly to Fe uptake is likely directly translated into benefits for Fe homeostasis and control of oxidative stress resulting from Fe uptake. Whether PATL2 also binds α-tocopherol in vivo remains unknown, as the vitamin is synthesized in plastids and would need to be exported. Further research into this direction could greatly contribute to our understanding of lipophilic antioxidants in plant cells and provide novel insight into the molecular operations of cellular metal ion uptake.

Under the presented premise, cells would recruit antioxidants directly to the site of entry, limiting oxidative damage to other molecules. This mechanism provides an elegant and safe solution for the conundrum of needing Fe but also needing to maintain a healthy cell. Given that Fe deficiency is one of the most wide-spread nutrient deficiencies in humans, the identification of this mechanism might help to increase Fe content in plant-based foods. Breeding plants with optimal nutritional value will be instrumental toward a sustainable food supply and help reduce the quantity and, therefore, the environmental impact of animal-derived products.
